# The Contribution of the Human Oral Microbiome to Oral Disease: A Review

**DOI:** 10.3390/microorganisms11020318

**Published:** 2023-01-26

**Authors:** Austin Gregory Morrison, Soumyadev Sarkar, Shahid Umar, Sonny T. M. Lee, Sufi Mary Thomas

**Affiliations:** 1 Department of Cancer Biology, University of Kansas Medical Center, Kansas City, KS 66160, USA; 2 Division of Biology, Kansas State University, Manhattan, KS 66506, USA; 3 Department of General Surgery, University of Kansas Medical Center, Kansas City, KS 66160, USA; 4 1717 Claflin Road, 136 Ackert Hall, Manhattan, KS 66506, USA; 5 Departments of Otolaryngology, University of Kansas Medical Center, Kansas City, KS 66160, USA; 6 Departments of Anatomy and Cell Biology, University of Kansas Medical Center, Kansas City, KS 66160, USA; 7 3901 Rainbow Blvd., 4031 Wahl Hall East, MS 3040, Kansas City, KS 66160, USA

**Keywords:** oral microbiome, oral cancer, oral disease, 16S rRNA gene sequencing, *Fusobacterium nucleatum*, *Porphyromonas gingivalis*, *Prevotella*, *Streptococcus mutans*, *Leptotrichia*

## Abstract

The oral microbiome is an emerging field that has been a topic of discussion since the development of next generation sequencing and the implementation of the human microbiome project. This article reviews the current literature surrounding the oral microbiome, briefly highlighting most recent methods of microbiome characterization including cutting edge omics, databases for the microbiome, and areas with current gaps in knowledge. This article also describes reports on microorganisms contained in the oral microbiome which include viruses, archaea, fungi, and bacteria, and provides an in-depth analysis of their significant roles in tissue homeostasis. Finally, we detail key bacteria involved in oral disease, including oral cancer, and the current research surrounding their role in stimulation of inflammatory cytokines, the role of gingival crevicular fluid in periodontal disease, the creation of a network of interactions between microorganisms, the influence of the planktonic microbiome and cospecies biofilms, and the implications of antibiotic resistance. This paper provides a comprehensive literature analysis while also identifying gaps in knowledge to enable future studies to be conducted.

## 1. Introduction

The human microbiome is defined as the entire genomic content of the microorganisms inhabiting a particular site of the human body. Nobel prize laureate Joshua Lederberg first coined the term “microbiome” as a community of commensal, symbiotic, and pathogenic microorganisms [1]. It has also been noted that these microorganisms share physical space within the human body [1]. It was initially thought of as common knowledge that bacteria present in the human body outnumbered human cells by 10:1; however, recent studies have shown that a more realistic ratio for bacteria to human cells is 1:1 [2]. The human microbiome exists in different ecological niches. This can include that of the oral cavity, skin surface, intestinal tract, esophagus, lungs, and others; all of which communicate and interact within the molecular microenvironment [3]. The terminology “microbiome” and “microbiota” are often used interchangeably; however, this is inaccurate. The term “microbiome” refers to the collective genomes of the microorganisms residing within a given environment, while the term “microbiota” refers to the wide variety of microorganisms that reside within a given environment. Emerging evidence suggests the importance of having a balanced microbiota to maintain proper digestion, metabolism, and immune response [4]. Of the human microbiome, the most widely studied is the gut microbiota. Digestive diseases that result in inflammation are often due to dysbiosis of the gut microbiota [5]. However, there is emerging data that dysbiosis in other areas of the human microbiome such as the pancreas, oral cavity, sinonasal cavity, and vaginal cavity can also cause disease [6,7]. It has recently been shown that local tumor microbiota influences survival independent of immune therapy, albeit the direct link to the microbiome is not understood, indicating a need for further exploration [8,9]. In this review, we focus on the oral microbiome in order to better understand its effects on periodontal disease.

The human oral microbiome is similarly defined as the entire genomic composition of the population of microbial organisms that resides in the oral cavity. The oral microbiota was first identified by Antony van Leeuwenhoek who observed his own dental plaque and described it as “little living animalcules prettily moving” [10,11]. He was later acknowledged as the father of microbiology, after being credited for discovering both protists and bacteria [12]. Today the human oral microbiome is still readily studied and it is now understood that there are a combination of microorganisms that are heavily colonized within the oral cavity including viruses, protozoa, fungi, archaea, and bacteria [13]. After the colon, the oral microbiota is the second most complex in terms of species [14]. Of these microorganisms, the most widely studied is bacteria. The oral microbiota resides in both saliva and the surface area of the mouth. The bacteriome of the saliva is dominated by the *Streptococcus*, *Prevotella*, and *Veillonella* genera, which comprise 70% of this microbiota [10]. Studies have shown that the neonatal microbiota may have a prenatal origin [15]. *Fusobacteria* is among some of the most common cultivable microorganisms found, but other pioneer bacterial populations include *Streptococcus*, *Lactobacillus*, *Actinomyces*, *Neisseria*, and *Veillonella* [11]. The oral surface cavity is about 214.7 ± 12.9 cm^2^, and there is no significant difference due to gender in this regard. The teeth, keratinized epithelium, and nonkeratinized epithelium occupy about 20%, 50%, and 30% of the total surface area, respectively [16]. The mouth cavity includes the inner cheeks, hard and soft palates, and tongue, with connections to the pharynx. Each of these surfaces, along with the saliva and gingival crevices, contain their own microenvironments and harbor site-specific microbiota [17]. Next generation DNA sequencing is allowing for a much deeper analysis; however, it may not be enough on its own to make conclusions because of the strong influence of the environment on heterogeneity across samples from both the same individual at different sites of the oral cavity (tongue, palate, teeth, cheeks, floor, etc.) and between individuals [5]. Some of the main considerations in using sequencing strategies to study the oral bacteriome are discussed later. Moreover, there are some biases related to PCR amplification errors and assembly strategies. This approach also requires high sequence coverage that has a direct relation with the high cost associated with this technique [18].

The normal microbiota of the oral cavity is associated with oral pathologies, some of which are involved with periodontal disease. Periodontal disease is caused by the sessile and planktonic microbiota found within the saliva and dental plaque. The migration of these microorganisms leads to inflammation of the gingiva [19]. This inflammation leads to increased flow of gingival crevicular fluid, an inflammatory exudate found in the periodontal pocket between the tooth and the marginal gingiva. This increased flow is thought to contribute to host defense by flushing away bacteria from the periodontal pocket [20]. Even though oral microbes are the foremost cause of periodontitis, other risk factors include tobacco use, osteoporosis, obesity, and diabetes. These risk factors in combination with the oral microbes intensify the disease [21]. Other more generalized diseases that have been correlated with oral disease include rheumatoid arthritis [22], endocarditis [23], bacteremia [23], cardiovascular disease [24], pulmonary disease [25], liver disease [26], and cancer (gastrointestinal, pancreatic, and breast) [27]. Microbial oral biofilms contain a complex microenvironment which protects oral microbes from UV light exposure, dehydration, host immune cells, and killing molecules. The biological and non-biological surface of these biofilms within the oral cavity provide a level of protection for the microbes, making biofilm mediated infection extremely dangerous and difficult to eliminate [28,29]. In recent years, evidence has emerged to show a relationship between periodontal disease and oral cancer. Patients with oral cancer often have inflammation and poor oral hygiene suggesting the possibility that the oral microbiota may play a role in carcinogenesis. Chronic inflammation has been linked to the development of cancer through the pro-inflammatory cytokine release [30]. In addition, the mucosal microenvironment is a specific niche that can genetically influence epithelial cells. In the saliva, poor dental hygiene increases production of carcinogenic acetaldehyde from ethanol [31]. The imbalance of the microbiome has been shown to effect immune cell activation, although the mechanisms in which this occurs are not well understood [32]. Head and neck squamous cell carcinoma (HNSCC) is the most frequently occurring malignancy of oral cavity cancers with an incidence of over 90% of all cancers. There are between 350,000–400,000 new cases each year [33]. With this elevated incidence coupled with an increase in cases each year, the human oral microbiome is an important subject of research.

## 2. The Human Microbiome Analysis

The human microbiome has now been studied over a span of several decades. Initially, microbiology was almost totally dependent on the ability to isolate a culture and grow that culture exponentially in a sterile environment to study it. The difficulties in this method were vast, in that unknown microbial species had to be identified and then grown on a specific selected medium to promote growth, or they were identified by either their morphological or metabolic characteristics [34]. Thus, the total microbiome composition was skewed to those which were cultivatable and more readily studied. This remained an issue until nucleic acid sequencing technology was first used in the 1980s. This technology, in combination with recombinant DNA technology, allowed for a more comprehensive study of mixed microbial populations [35]. As technology has continued to improve over the last few decades, it has allowed the scientific community to have a more accurate understanding of the diversity of microbial species found within the human body.

Advancements in technology have also led to an increased understanding of the human microbiome. The following two methods are commonly used to study the microbiome: a targeted gene approach and a metagenomics approach. The latter is sometimes referred to as a shotgun approach because of the randomness in which genomic data is acquired [36]. Both of these methods utilize next generation sequencing as their main platform of analysis. The advantages in using a targeted gene approach is that it is cost-effective, the use of this analysis is widely accepted due to extensive research to compare to with popular databases, protocols for bioinformatic analysis have been well established, and host contamination is much more easily resolved. However, a lack of functional information and PCR bias can cause discrepancies in the differentiation of taxa [37]. The advantage in using shotgun metagenomics is that it encompasses all the microbial genomes present, including the host genome. Alternatively, this can also be viewed as a weakness due to its lack of specificity. Other disadvantages include the following: the cost of the computationally vast analysis, lack of well-established protocols, and the lack of consensus on analytical pipelines due to deficiencies in databases to make comparisons [37]. An overview of the processes can be seen in Table 1.

The ribosome is responsible for the translation of RNA into proteins. The 16S RNA is a gene present only in bacteria. It is found at the level of the ribosomes; these are formed of two subunits allowing the translation of RNA into proteins. The targeted gene approach to study the microbiome incorporates the use of the 16S ribosomal RNA gene sequencing for bacteria. Studies using this approach also typically involve using one or a combination of multiple hypervariable regions. Within bacterial 16S RNA, there are nine hypervariable regions (V1–V9). These regions are flanked by conserved regions in most bacteria [43]. This allows for more specificity when determining the phylogenetic assignment of bacteria. Evolution has led to variations in genes over time, which allows a unique fingerprint to be assigned to individual taxonomies and members of the microbial community. Due to its slow evolution, only a marginal number of bacterial genomes include identical 16S rRNA sequences, and this diversity rises with increasing 16S copy numbers [44]. However, during the amplification of each individual gene, a PCR product is sequenced one single time which allows for multiple chances of error to occur [37,45]. This potentiated the need to bundle sequences based on similarity to construct operational taxonomic units (OTUs) by using opensource software like Mothur, and more recently the construction of Amplicon sequence variants (ASVs) by using databases such as Quantitative Insights into Microbial Ecology 2 (QIIME2) which was launched in January of 2019. This imposed the need to make certain assumptions, such as the sequences >95% identity represent the same genus and the sequences >97% identity represent the same family [46]. Taxonomy can be assigned through data analysis by mapping to reference databases such as Silva and EzBioCloud. The classification of bacteria is established phylogenetically. It can be arranged in a hierarchy as follows: Domain, Kingdom, Phylum, Class, Order, Family, Genus, Species [47]. When sequencing fungi, the internal transcribed spacer (ITS) region is utilized. The ITS region exists between 16S and 23S rRNA genes and when this region is targeted for species characterization, it allows for the greatest differentiation and delineation of systematic relationships [48]. The most commonly used database for molecular identification of fungi is UNITE [49].

Shotgun metagenomics consists of an untargeted sequencing of the microbial genomes which contains a wide variety of genetic information that can be useful in the study of microorganisms. Currently, there are two strategies to obtain metagenome-assembled genomes. In the absence of any available reference genomes, the de novo option can be used where the sequence reads are compared against each other to ensure reads overlapping that leads to the generation of longer contiguous sequences (contigs) [50]. Another option is the reference-based assembly approach [51] in which reads are mapped to the reference genomes and is capable of detecting single nucleotide polymorphisms (SNPs), identification of potential functions, etc. [52]. The process for shotgun metagenomics is much more time consuming than that of targeted gene sequencing. At every step of the process, there are multiple considerations in order to ensure reproducibility and integrity of the data. There are currently multiple DNA extraction techniques for metagenomics experiments. Each one of these has its pros and cons and can cause variations in generating the raw reads. There have been reports that when different protocols have been applied it has resulted in variable bacterial taxa distributions [53]. So, it is always advisable to standardize a fixed protocol for a specific type of sample using a pilot study. Whatever the approaches used, there is still a chance of unwanted sequences being generated and proper steps should be taken, especially during sample preparation, to remove unwanted contaminations in order to bring out the targeted sequence [54]. Sometimes, there might be contaminations in the reagents and buffers of the kits themselves and these contaminations have been found to vary with different batches [55]. Previous studies have even reported the presence of microbes in kits, thereby contaminating the populations of study [56]. The use of different batches influencing the metagenomics workflow can be negated by a combination of using proper reagent blanks and ensuring random processing of samples. Thus, it is critical to ensure that the best kit is selected as well as the samples are randomly processed when used in multiple batches for optimum results [57].

Often it is the case that certain segments of the genomes remain underrepresented after sequencing. The primary reasons on which sequencing coverage and quality depends are (a) sequencing depth; (b) sequencing technology; (c) complexity of the genome [58,59]. So, implementing a correct balance between these three parameters leads to a successful execution of the study. There are ways to calculate the sequencing depth for a study based on the taxonomic profile of the community, but there is never a strict rule of thumb [38]. Ideally, the sequencing depth should be decided based on the research question, experimental design, and budget. Illumina’s sequencers ensure a high read quality coupled with the lowest per-based pricing and lead the sequencing market as it is a more reasonable option than PacBio and Oxford Nanopore [60]. However, the PacBio and Nanopore sequencing technologies are ever-evolving and provide a long-read sequencing approach which might be important to specific studies, and thus possess great potential [60].

Once the sequencing is completed, preprocessing of the sequence reads is required before analysis can begin to build the taxonomy profile. This process is filled with multiple experimental and computational approaches which lead to the lack of consensus on specific analytical pipelines [39]. Metagenomics data analysis is an active subject of research and is being updated almost every week as we are far from standardizing the most optimal one [60]. At present, the most accepted workflow typically follows the following steps: (a) initial quality control; (b) using tools such as MetaPhlAn to assign taxonomy to individual reads to generate community taxonomic composition [40]; (c) searching the reads against databases like KEGG can yield functional composition using tools such as MEGAN [41], MG-RAST [42], HUMAnN [61]. Another approach uses Meta_IDBA assemblers-based genome assembly [62], after which CONCOCT [63] can be applied to bin contigs into groups. Then, Anvi’o [64] can be used to perform the binning correction followed by subsequent annotations and functional analysis. In terms of reference-based metagenomic analyses, several software, including MetaCompass is a software package that allows for reference-assisted assembly of metagenomic data of low abundance genomes [65]. This is done by mapping reads against reference data sets, constructing reference-assisted contigs, and then correcting the assemblies by using Pilon [66]. This is limited by the availability of reference genomes and databases used for cross-referencing. For an unambiguous identification of microbial species from sequencing data, the electronic Tree of Life (eTOL) can be used. This allows for the referencing of human tissues across the entire tree of life, including archaea, bacteria, chloroplasts, basal eukaryotes, fungi, and holozoan. The use of eTOL is especially important for viral sequences due to significant homology between human sequences. This can be combatted using a ‘stripping’ method where similar sequences are removed from the viral genome before characterization [67].

A less common method of characterization of members of the human microbiome is metabolomics. Metabolomics is a comprehensive approach where all the metabolites of a sample are included in the analysis [68]. The advantage of metabolomics is that small intermediate molecules can be used to identify metabolites through mass spectrometry coupled with chromatographic separation techniques. A direct measurement of the underlying biochemical activity can provide information to help identify the origin of the metabolite. This provides information on the host metabolism as well [69]. Metabolomics provides a more comprehensive view of cell function, which has been seen as bridging the gap between genotype and phenotype [70]. Another emerging method of analysis is metaproteomics which similarly uses mass spectrometry; however, the main focus is to identify proteins present within the microbiome [37,71]. With metabolomic and proteomic analysis, there is a lack of comprehensive datasets when compared to nucleic acid-based methods regarding microorganisms. Metatranscriptomics is the study of gene expression of microbes within their natural environment. This helps to provide information about the active functions of microbial communities, information that is not discovered from a simple microbe composition. However, with metatranscriptomics, sample collection is destructive and experimental design is critical [72]. The use of transcriptomics and proteomics also only accounts for the biological potential and ignores the cellular activity component. This can be overcome through the use of metabolomics to reflect the enzymatic activity of the cell. Microbial culturomics is another method that has started to gain popularity within the last few years. This method combines the culturing conditions of unknown bacteria with popular identification methods to determine their significance within the human microbiome [73]. While these methods are less common than those mentioned previously, new developments in the field of bioinformatics continue to push the edge on ways to characterize the human microbiome. All methods are highlighted in Table 1.

The Human Microbiome Project (HMP) was a project launched by the NIH in October 2007, shortly after the completion of the Human Genome Project. There was increasing interest in the scientific community to fully sequence the genome of microbes growing within the human body [74]. The NIH defined the goal of the project as “specifically devised and implemented to create a set of data, reagents, or other material whose primary utility will be as a resource for the broad scientific community” [75]. Initially, this project was supposed to be completed within five years. However, after discovering that the correlation between the host phenotype and taxonomic composition of the microbiome was often not sufficient [14], a second phase of the project was initiated. The Integrative HMP was designed to study host–microbiome interactions, including immunity, metabolism, and dynamic molecular activity [76]. Although work is still continuing in this area, one advantageous aspect has been the creation of online databases to expedite characterization of the human microbiome.

## 3. The Human Oral Microbiome

The human mouth is home to a vast variety of microorganisms that rely on each other to maintain stable conditions. For the most part, these microorganisms live in harmony with one another and the host. In part, this symbiosis is due to the resident commensal flora, including planktonic bacteria, that occupies the hard and mucous surfaces preventing the establishment of exogenous microorganisms. When last updated, the expanded Human Oral Microbiome Database (eHOMD, www.homd.org (accessed on 9 December 2022)) included a total of 772 microbial species present in the human aerodigestive tract [77]. Of those species, 57% are cultivated and named, 13% are cultivated but unnamed, and 30% are uncultivated. A total of 1570 genomes from 475 taxa can be viewed in the genome browser software. The composition of the oral microbiome includes viruses, archaea, fungi, and bacteria. The most common identification tool used for characterizing the oral bacteriome is 16S rRNA amplicon sequencing by targeted gene approach, as listed previously [78]. However, sample collection can be difficult as there is no standardized procedure for collecting oral microbiome samples. Several parameters can vary the collection, including timing of the day, before or after meals, the sample collection tools, and the storage conditions of the samples. Similar to the sample collection process, there is a lack of standardization for the sequencing analysis as well. For these reasons, it can be difficult to make reliable assumptions from the data.

A substantial number of viruses co-exist in the oral cavity, most of which are considered pathogens. Viruses and phages outnumber all other biological entities on earth; however, the virome, that is the viral component of the microbiome, has not been studied well enough to conclude its overall diversity in the mouth [79]. Recent focus has been on the bacteria and its role in the oral microbiome, leaving the effects of viruses in the microbiome vastly understudied [80]. As technological advancements are discovered, new information is brought to light debunking ideas that were once thought of as common knowledge. Human blood was once thought of as aseptic, but it is now known that it contains both viruses and phages [79]. Phage, or bacteriophage, are viruses that infect and replicate within bacteria. This can be done in one of these two ways: the lytic cycle where the phage uses bacterial processes to replicate and then lyses the bacterial cells, or the lysogenic cycle where the phage inserts its DNA into the bacterial chromosome to be replicated. A large number of phages have been shown to be a main component of the oral virome. It has been shown that a majority of virus sequences found within the oral cavity had homology with phages, suggesting a predominant role in lysogeny, as this is one of the two ways previously mentioned for phages to infect bacteria [81]. Hepatitis viruses and HIV are examples of viruses that enter through the oral cavity by traversing the oral mucosa and travel into the upper respiratory tract, causing the immune system to become compromised [79,82,83]. Human papilloma virus (HPV) has been shown to not only cause oral disease, but also to be a dominant microbial factor in HNSCC [84]. Herpes simplex virus has also been shown to cause gingival lesions [85]. With the overwhelming presence of viruses in the oral cavity, it is not surprising that the virome plays a major role in oral disease.

The *Archaea* are found to play a minor role in the oral microbiome; however, due to the diversity of the community that makes up the oral microbiome, and the lack of understanding of the interrelationships between microorganisms, *Archaea* should not be considered nonessential at this time. All of the members of the archaeal community found within the oral cavity are considered methanogens, meaning they obtain most of their metabolic energy from the biosynthesis of methane [13]. *Archaea* are increased in periodontitis subjects with *Methanobrevibacter oralis*, *Methanobacterium curvum/congolense*, and *Methanosarcina mazeii* showing the greatest prevalence [86]. *Archea* may be contributing to this diseased state through interspecies hydrogen transfer and by favoring the growth of fermenting bacteria. Recently, there has been a correlation identified between methanogens and colorectal cancer [87]. Methanogens possess the ability to transfer heavy metals into highly toxic methylated derivatives. Continued research needs to be performed in order to better understand the interrelationships between *Archaea* and other microorganisms to determine the more accurate role that these organisms play in oral disease.

The fungi that reside in the microbiome are collectively known as the mycobiome, which is an emerging topic of discussion. During a study of the oral cavity of 20 healthy individuals, the “basal” oral mycobiome was shown to contain 85 genera—74 culturable and 11 non-culturable [88]. More recent studies show over 100 culturable species of fungi in the oral cavity alone [89]. Current topics of research have focused around the fungal species *Candida albicans* (*C. albicans*) which is commonly found in the oral mucosa but can rapidly take on pathogenic properties [90]. Some of these properties include stimulating invasive filamentous growth in a hypoxic environment, and maintaining continued growth in an environment with minimal nutrition by inducing alternative metabolic pathways like the glyoxylate cycle, gluconeogenesis, and fatty acid oxidation [91,92]. Other members of the oral microbiome, such as streptococci, have been shown to provide advantageous elements, for instance a carbon source and an adhesion site, for *C. albicans* to flourish [90]. This may not be so advantageous to the host however, as studies in a rat model have shown that in combination, *Streptococcus mutans* (*S. mutans*), a known carcinogen, and *C. albicans* heightens the development of dental caries [93]. These interactions have shown the possibility of *C. albicans* acting as a bridge for bacteria, allowing them to adhere to mucosal surfaces [94,95]. Conversely, *Streptococcus salivarius* has been shown to prevent the adherence of *C. albicans* to mucosal surfaces, suggesting the potential for use as a probiotic [96]. Mechanisms for defense have developed; human β-defensins mediate this cross-talk and help to maintain a healthy oral mucosal environment [97]. Both an early and late host immune response occurs when *C. albicans* is present in pathogenic levels. There is early recognition by neutrophils, macrophages, and dendritic cells, but there is also an adaptive antifungal response [98]. The combination of all these factors represents the importance of the mycobiome and its relevance within the oral microbiome.

The most substantial contributor to the oral microbiome, and the most broadly studied, is bacterium. This composition of bacteria is fairly conserved among several factors including age, race, sex, and geographical location. A study of 120 individuals from 12 geographic locations determined that there was no significant geographical variance between individuals’ salivary microbiota [10,13]. There are six major phyla, accounting for 96% of the taxa, that constitute the oral microbiome—*Firmicutes*, *Bacteroidetes*, *Proteobacteria*, *Actinobacteria*, *Spirochaetes*, *and Fusobacteria.* The remaining 4% of the taxa are made up by the phyla *Euryarchaeota*, *Chlamydia*, *Chloroflexi*, SR1, *Synergistetes*, *Tenericutes*, and *Saccharibacteria* [99,100]. While these are all commensal in nature, some of the bacteria from these phyla have also been viewed as pathogens. The transition from commensal to pathological most often depends on the quantity of this microorganism within the biofilms of the oral cavity. Two of the most significant bacteria to harbor pathogenic properties are *F. nucleatum* and *Porphyromonas gingivalis* (*P. gingivalis*) [101]. Dysbiosis was reported in e-cigarette users compared with never smokers or tobacco cigarette smokers. *Porphyromonas* and *Veillonella* were higher among vapers [102]. As previously mentioned *S. mutans* is a known carcinogenic bacterium observed during the formation of dental caries [93]. Several species from the genus *Prevotella* have been implicated in periodontitis including *P. dentalis*, *P. enoeca*, *P. fusca*, *P. melaninogenica*, *P. denticola*, *P. intermedia 17*, *P. intermedia 17-2* [103]. *Leptotrichia buccalis* (*L. buccalis*) has been isolated and recovered from patients with varying levels of gingivitis [104]. A majority of the bacteria in the oral microbiome are strict anaerobes, making them more difficult to culture and thus requiring extreme caution when handling and processing samples [90]. These bacteria will be reviewed in further detail in the ensuing section. It is obvious from the extensive characterization of the bacterial taxa in the oral microbiome that they are central players involved in both oral and overall health. Considerable studies need to be conducted in order to identify better avenues to counter the detrimental effects of these bacteria.

## 4. Key Bacteria Involved in Oral Disease

*L. buccalis* is a Gram-negative, rod-shaped facultative anaerobic bacterium that is a normal resident of the oral flora. It is placed in the phylum *Fusobacteria* in the family *Leptotrichiaceae*. Growth can be visualized by streaking bacteria onto a blood agar plate under anaerobic conditions. Members of the *Leptotrichia* genus are similar in size and shape so identification is typically performed through 16S rRNA gene sequencing [104]. *Leptotrichia* survives off the fermentation of carbohydrates, ultimately producing lactic acid as an end product [105]. Studies have shown that *L. buccalis*, recovered from patients with gingivitis, may play a role in the inflammatory process [104], by regulating expression of pro-inflammatory interleukin (IL)-1β, IL-6, IL-8 and the anti-inflammatory IL-10 [106]. While it is possible that *Leptotrichia* plays a direct role in the inflammatory process, a vast number of oral bacteria has been implicated in the inflammatory response pathways, so more than likely there is a combination of microorganisms involved. Ultimately, further studies need to be conducted in order to determine the true role that each of the key bacteria plays.

The genus *Prevotella* and several of its species have been known to cause disease in the oral cavity. Some of the species that are involved include *P. dentalis*, *P. enoeca*, *P. fusca*, *P. melaninogenica*, *P. denticola*, *P. intermedia 17*, *P. intermedia 17-2* [103]. *Prevotella* are gram-negative, anaerobic bacteria that are common members of the oral microbiome. They are placed in the phylum *Bacteroidetes* in the family *Prevotellaceae*. *Prevotella* has also been observed as a commensal in both vaginal microbiota and gut microbiota [107,108]. *Prevotella* initiates the production of Toll-like receptor (TLR) 2, which subsequently leads to the polarization of Th17 while production of inflammatory cytokines can lead to increased prevalence in periodontal disease [103]. Studies in mice have also shown *Prevotella* to promote clinical features of human disease. An upregulation of *Prevotella* and a downregulation of *Lactobacillus*, a major member of the lactic acid bacteria group, have shown correlation to osteomyelitis in mice. When *Prevotella* is reduced and *Lactobacillus* is increased, protection against osteomyelitis has been observed [109]. A continuation of research into *Prevotella* could show the clinical importance of its participation in oral disease.

*Helicobacter pylori* (*H. pylori*) is a Gram-negative, microaerophilic, helical bacterium that is commonly found in the stomach. *H. pylori* has been implicated in a variety of diseases including duodenal ulcers, gastric ulcers, gastric atrophy, gastritis, and gastric carcinoma [110]. Recently, there has been much controversy surrounding whether *H. pylori* also frequently co-habitats within the oral cavity or only during disease states [111]. The helical shape of *H. pylori* is thought to have evolved in order to better help it penetrate the mucosal lining of the stomach [112]. Having once been considered a species of *Campylobacter*, it is similar in nature to their corkscrew shapes. Once infected, removal of *H. pylori* from the oral cavity is much more difficult than the eradication from the stomach. Typically, antibacterial therapy is used to remove the bacterium. However, if this is ineffective, *H. pylori* will reinfect the stomach within a couple of weeks [113]. In addition, studies have found that improved oral health and hygiene are unlikely to increase the efficacy of *H. pylori* eradication from the stomach [114]. In contrast, long-term dental plaque control has been implicated to help prevent *H. plyori*-induced gastric disease or re-infection [115], suggesting a need for further research into the relationship of oral health and *H. pylori.*

*S. mutans* is a gram-positive, facultatively anaerobic bacterium found within the oral microbiome. It is located within different areas of the oral cavity, but the round bacterium most commonly inhabits the pits and fissures. This known carcinogen has recently been considered an opportunistic commensal of the oral cavity, due to its interactions with certain fungal species. This communication between *S. mutans* and fungal species allows the microorganisms to exchange chemical signals, known as quorum sensing, which help to promote the formation of oral biofilms [116]. The combination of *S. mutans* and *C. albicans* has often been considered the main contributor to dental caries, specifically early childhood caries (ECC) [117]. The bacterial etiology of ECC is complicated due to the colonizing of bacteria while the teeth are forming and being susceptible to demineralization [118]. Cospecies biofilms accumulate more biomass and increase the likelihood for periodontal disease [117]. Dental caries are still considered the most common chronic disease worldwide, and there are currently very few approved precision antibiotic treatments to prevent them. However, recent studies have shown the potential for synthetic molecules paired with antibiotics to target specific domains of the dental caries [119]. Other studies have shown promising results using *Lactobacillus salivarius* probiotics to reduce pathogenic species and biofilm mass, although future research will aim to look at the long-term effects of method [120]. Other future studies will look to push the edge on how dental caries are controlled on a worldwide basis.

One of the most substantial bacteria involved in periodontal disease is *P. gingivalis* [121,122]. This bacterium is a Gram-negative, rod-shaped, anaerobe that belongs to the phylum *Bacteroidetes* and the family *Porphyromonadaceae. P. gingivalis* is a known pathogen that dwells in the oral cavity and can be visualized on blood agar, forming black colonies. This bacterium thrives off a dysbiotic environment in the oral microbiome. In order to achieve this dysbiosis, *P. gingivalis* manipulates complement-TLR crosstalk which activates an inflammatory response. The combination of dysbiosis with inflammation leads to a situation in which periodontal disease blooms [123]. Studies have shown that in vitro *P. gingivalis* can invade human fibroblasts and be able to achieve a level of antibiotic resistance [124]. Most strains of *P. gingivalis* have been shown to exhibit fimbriae, or long and thin proteinaceous surface appendages which play essential roles in the binding and invasion of host cells [125]. One study declared that fimbriae type II has the ability to bind to α_5_β_1_-integrin, which facilitates bacteria to be taken up by phagocytes and dendritic cells further permitting the rearrangement of the actin cytoskeleton [126]. *P. gingivalis* infection has also been shown to increase the risk of developing oral and digestive cancers, and this bacterium is frequently found in oral squamous cell carcinoma [127]. This bacterium promotes PI3K/Akt signaling which leads to host cell proliferation by reducing apoptosis. Tumorigenesis is further promoted through *P. gingivalis*-facilitated EMT because of GSK3-beta downregulation, reduced E-cadherin, and increased pro-MMP9. Additionally, this bacterium stimulates tumor growth and metastasis by inhibiting p53 [128]. A combination of these factors makes *P. gingivalis* a deadly pathogen that causes widespread periodontal disease and increased risk of oral cancer simply by its presence.

**Figure 1:**
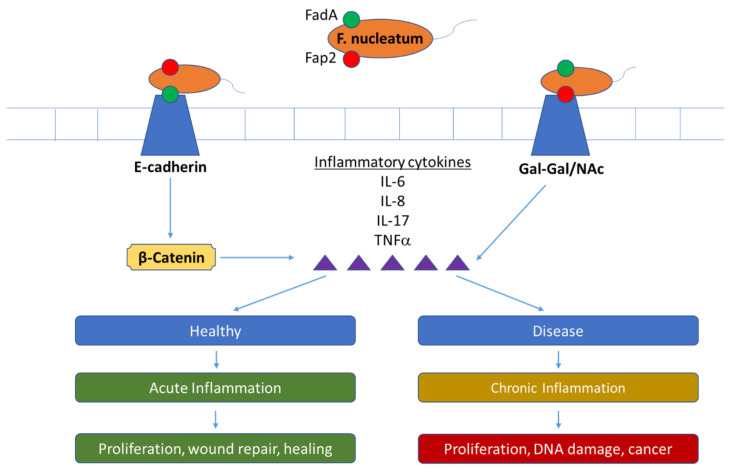
The role of *Fusobacterium nucleatum* in the inflammation and ultimately the increase in cell proliferation of host cells. Both FadA and Fap2 are encoded by *F. nucleatum*, and their individual pathways are also shown [129]. FadA utilizes the E-cadherin complex while Fap2 adheres to the galactose sensitive pathway through the use of N-acetylgalactosamine (Gal/NAc) [131]. *F. nucleatum* provokes a variety of host responses including the stimulation of IL-6, IL-8, IL-17, and TNF α [138]. During a healthy state, this inflammation leads to wound repair and healing. However, during a diseased state, this can lead to DNA damage and cancer.

Potentially the most influential bacterium implicated in periodontal disease is *F. nucleatum* [121,129]. This Gram-negative, anaerobic bacterium was once considered an oral commensal but is now more frequently referred to as a pathogen. *F. nucleatum* can be observed through growth in anaerobic conditions on a blood agar plate. *F. nucleatum* belongs to the phylum *Fusobacteria* and the family *Fusobacteriaceae.* This bacterium is one of the most abundant species in the oral cavity in both healthy and diseased individuals [130]. *F. nucleatum* was once thought to exist almost exclusively in the oral cavity [131]; however, under disease conditions, it can be found in extra-oral sites and has been implicated in diseases such as inflammatory bowel disease, colorectal cancer, Crohn’s disease, rheumatoid arthritis, meningitis, appendicitis, and adverse pregnancy outcomes [132,133,134,135,136,137]. When this species is found in abundance in the oral cavity, or located in extra-oral sites, it has been known to exhibit pathogenic properties [129]. Studies have shown that *F. nucleatum* can increase tumor growth by activating the IL-6-STAT3 axis through the signaling of Fusobacterium Adhesin A (FadA) [138]. Figure 1 provides the pathway in which FadA is involved. Other bacteria with known pathogenic roles in periodontal disease, such as genera *Leptotrichia*, *Prevotella*, *Streptococcus*, and *Porphyromonas* will also be reviewed to highlight the importance of their continued study.

In co-culture, *F. nucelatum* is considered a secondary colonizer after primary colonizers, such as *Streptococcus*, and is vital in the structural relationships of the polymicrobial biofilm [139]. *F. nucleatum* encodes for the *fadA* gene which is an adhesin that binds to host cells and is required for the binding of normal and cancerous cells, suggesting a potential mechanism for the invasion of anti-tumor immune response [129]. Fap2 is a galactose sensitive adhesin that is involved in cell adhesion and the inflammatory pathway [140]. *F. nucleatum* provokes a variety of host responses including the stimulation of IL-6, IL-8, and TNF α [141]. Inflammatory responses involved in periodontal disease are activated by the binding of *F. nucleatum* to natural killer cells [142]. These pathways can be better visualized in the schematic provided in Figure 1. The diseases where *F. nucleatum* is implicated, have wide variation, from mild reversible forms of gingivitis to generalized aggressive forms of periodontitis [143]. *F. nucleatum* has been found in a variety of extra oral sites [131], and it is speculated that FadA binding to cadherins on epithelial and endothelial cells is likely the reason for colonization on multiple body sites [129].

Recent evidence has emerged showing the potential for antibiotic resistance via *F. nucleatum* and *Pseudomonas aeruginosa* interaction through FadA [144]. Another recent study observed the targeting of TLR4 and specific microRNAs to activate the autophagy pathway and change the chemotherapeutic response, promoting chemoresistance [145]. Further studies have investigated the role of *F. nucleatum* and *P. gingivalis* in oral cancer development and progression in mice models. These bacteria are often found together in oral cancer cells triggering the release IL-6, activating the STAT3 pathway. This increase in pSTAT3 and IL-6 is also greater in mice infected with *F. nucleatum* and *P. gingivalis* than in non-infected mice [146]. With all of the implications in disease, both major and minor, *F. nucleatum* will certainly be a topic of future discussion.

## 5. Conclusions

The oral microbiome is a vast environment that is made up of multiple species of microorganisms that include viruses, archaea, fungi, and bacteria. The development of 16S rRNA gene sequencing has enabled the characterization of the oral bacteriome, providing us with knowledge that would not have been obtainable only a few decades ago. However, this has left us with numerous questions and the curiosity to study not only the individual microorganisms, but also how they interact with one another. The oral microbiome is an environment full of symbiotic relationships, but when pathogens interrupt this relationship and cause dysbiosis, a once healthy situation can become diseased and increase the likelihood of comorbidities. In recent years, science has been able to uncover some of the common properties that these pathogens share, such as the stimulation of inflammatory cytokines. It has also been implicated that certain bacteria can serve as a bridge for other invasive pathogens allowing for easier migration, however more research needs to be conducted to achieve the knowledge of both the reason for this and the mechanism by which this occurs. In this review, we summarized the current literature and highlighted the tools used to characterize the oral microbiome, while also mentioning the discrepancies within those techniques. We also provided a critical summary of key bacteria currently being studied, while suggesting gaps in knowledge for future studies. The study of the oral microbiome is one that has been around for the last few decades, nonetheless it is still very much considered as emerging. More precisely, the role of viruses, fungi and archaea remains poorly studied; it is to be expected that in the years to come, answers to these questions will be provided and knowledge on subjects which have not yet been addressed will be advanced.

## Figures and Tables

**Table 1:** A comprehensive description of the main microbiome characterization methods and applications.

Characterization Method	Description	References
Targeted Gene Analysis	Analysis involving the use of one or a couple hypervariable regions. This is the most common analysis and is best used for highly specific sequencing.	[26,27,28]
Shotgun Metagenomics	A widespread, untargeted approach that incorporates a wide variety of genetic information. This analysis is best used in the absence of reference genome.	[33,34,35]
Metabolomics	A comprehensive analysis which utilizes mass spectrometry for characterization. This is best used to gain information on the role of small metabolites in cell function.	[38,39]
Metraproteomics	Analysis using mass spectrometry to provide information on macromolecules. This should be used when inquiring about protein interaction with the system.	[26,40]
Metratransciptomics	An approach which utilizes microbes within their natural environment to provide additional information on the overall function of the community	[41]
Microbial Culturomics	A method used in conjunction with other characterization methods to provide information of unknown species through their culturing methods. Experimental design is critical.	[42]

## Data Availability

No new data were created or analyzed in this study. Data sharing is not applicable to this article.
